# The impact of primary total hip and knee replacement on frailty: an observational prospective analysis

**DOI:** 10.1186/s12891-024-07210-w

**Published:** 2024-01-20

**Authors:** Tobias Kappenschneider, Philip Bammert, Günther Maderbacher, Felix Greimel, Lukas Parik, Dominik Emanuel Holzapfel, Amadeus Dominik Schraag, Julia Götz, Katrin Michalk, Joachim Grifka, Matthias Meyer

**Affiliations:** 1 Department of Orthopaedic Surgery, Regensburg University Medical Center, Kaiser-Karl V.-Allee 3, 93077 Bad Abbach, Germany; 2 Department of Health Economics, Technical University of Munich, Munich, Germany

**Keywords:** Orthogeriatric, Frailty, Hip arthroplasty, Knee arthroplasty, Older people

## Abstract

**Background:**

Osteoarthritis is a prevalent condition in frail older adults that requires hip or knee replacement in many patients. The aim of the study was to determine the impact of hip and knee arthroplasty on frailty.

**Methods:**

In this prospective short-term study, we used data from 101 participants of the ongoing Special Orthopaedic Geriatrics (SOG) trial, funded by the German Federal Joint Committee (GBA). Frailty, measured by Fried’s Physical Frailty Phenotype (PFP), was assessed preoperatively, 7 days postoperatively, 4–6 weeks and 3 months after hip and knee arthroplasty. ANOVA with repeated measures and post-hoc tests for the subgroups were used for the statistical analysis.

**Results:**

Of the 101 participants, 50 were pre-frail (1–2 PFP criteria) and 51 were frail (≥ 3 PFP criteria) preoperatively. In the pre-frail group, the PFP score decreased from 1.56 ± 0.50 (median 2) preoperatively to 0.53 ± 0.73 (median 0) 3 months after surgery (*p* < 0.001). The PFP score in the frail cohort decreased from 3.39 ± 1.45 (median 3) preoperatively to 1.27 ± 1.14 (median 1) 3 months postoperatively (*p* < 0.001). While the PFP score of the pre-frail participants increased 7 days after surgery, the PFP score of the frail group decreased significantly.

**Conclusion:**

Pre-frail individuals often regain robustness and patients with frailty are no longer assessed as frail after surgery. Joint replacement is an effective intervention to improve frailty in hip and knee osteoarthritis.

**Trial registration:**

This study is part of the Special Orthopaedic Geriatrics (SOG) trial, German Clinical Trials Register DRKS00024102. Registered on 19 January 2021.

**Supplementary Information:**

The online version contains supplementary material available at 10.1186/s12891-024-07210-w.

## Background

Frailty is a multidimensional geriatric syndrome characterised by loss of individual reserve capacity and increased vulnerability to internal and external stressors [1]. A frail person is at increased risk of adverse outcomes such as falls, disability, hospitalisation and mortality [2, 3]. Therefore, frailty is an emerging global health burden that has significant implications for clinical practice and public health [2, 4]. A commonly used tool for assessing frailty is the Fried Frailty Phenotype [3]. This clinical phenotype of frailty manifests as multi-system pathology characterised by low physical activity, global weakness with low muscle strength, exhaustion, reduced walking speed and weight loss. Pre-frailty occurs at an earlier stage of the frailty spectrum and is associated with the later development of frailty [3, 5].

So far, many influencing factors have been identified that can promote the development of frailty. These include risk factors such as high age, multimorbidity, obesity, polypharmacy, low physical activity, and inflammation [1]. There is also an association of osteoarthritis (OA) with frailty and pre-frailty in older adults [6]. OA is considered the most prevalent chronic joint disease in the world [7]. Chronic comorbidities can lead to a progression of frailty [8]. However, little is known about the regression or reversibility of frailty. Interventions have so far been limited to physical training, high-protein diets, or a combination of both [9].

In Germany, total hip (THA) and knee arthroplasty (TKA) are among the 20 most common surgical procedures for hospitalised patients overall [10]. The aim of the study was to determine the impact of primary total hip and knee arthroplasty in elderly patients with OA on the Fried Frailty Phenotype. We hypothesised that hip and knee replacement would correlate with a decrease in frailty and pre-frailty as assessed by Fried’s phenotype criteria. The association of hip and knee OA with frailty has been clearly demonstrated and was also reconfirmed by the European Project on OSteoArthritis (EPOSA) in 2015 [6]. There is also no doubt that frailty can have an impact. Regression has been shown multiple times in studies involving physical activity and a high-protein diet or a combination of both [9]. It therefore stands to reason that if the trigger OA for the frailty is removed, i.e. the degenerated hip or knee joint is replaced and thus the cause eliminated, a regression or reversibility of the pre-frailty/frailty can also occur. The reason is to restore joint function and thus build muscle. As frailty is a multidimensional syndrome, factors such as improvement in pain and walking problems must also be considered. However, these are assessed directly or indirectly by the frailty phenotype criteria [3].

The baseline characteristics listed in Table 1 were selected because they are commonly used assessment tools in geriatrics, both clinically and scientifically, and are well descriptive of the population studied.

## Methods

### Study design

This study is part of the ongoing Special Orthopaedic Geriatrics (SOG) trial (German Clinical Trials Register, 19/01/2021, DRKS00024102). The SOG study is a monocentric, prospective, randomised controlled trial funded by the German Federal Joint Committee (GBA). The original study aimed to investigate a specially developed multimodal care model (SOG care model) for orthogeriatric patients with total hip and total knee arthroplasty compared to usual orthopaedic care without orthogeriatric co-management. Frailty was a secondary outcome measure. A detailed description of the study can be found elsewhere [11]. The current study enrolled all 125 patients who underwent surgery in the SOG trial between 01 April 2021 and 30 November 2022. This additional analysis was not planned when the original study was designed.

### Data collection

In the Orthopaedic Department of the University Hospital of Regensburg, about 18,000 patients are treated annually in the university outpatient clinic and more than 1500 endoprosthetic procedures on hip and knee joints are performed each year. Participants were recruited at the university outpatient clinic if they were diagnosed with primary hip or knee osteoarthritis and had an indication for THA or TKA. The study data were collected preoperatively, on the 7th day after surgery before discharge, 4–6 weeks, and 3 months after surgery.

### Study population

Eligibility criteria included: primary hip or knee osteoarthritis, age 70 years and older with multimorbidity or age 80 years and older, indication for elective unilateral hip or knee replacement and pre-frailty or frailty according to Fried’s criteria [3]. Exclusion criteria were age under 70 years, previous bony surgery or tumour in the area of the joint to be treated, acute infection, robustness (0 criteria according to Fried’s Frailty Phenotype) [3] and increased need for care (care level ≥ 4; severe impairment of independence, need for help with basic care 24 h a day).

Out of a total of 125 subjects in the SOG study, there were 7 drop-outs. The reasons were cancellation of surgery or refusal to participate in the study. Another 17 patients were excluded due to robustness (0 criteria). As a result, the number of people included in the analysis was 101. The number of patients lost to follow-up was 3 at 4–6 weeks (follow-up 1) and 1 at 3 months (follow-up 2).

### Surgical techniques and implants

All operations were performed in a single Department of Orthopaedic Surgery of a University Medical Centre. The lateral decubitus position was used for the cementless THA. A minimally invasive anterolateral approach was chosen [12]. Press-fit acetabular components and cementless stems from a single manufacturer (Pinnacle cup, Corail or Trilock stem; DePuy, Warsaw, IN) were used in all THAs. The cemented TKA was performed via a medial parapatellar approach. Cemented components from a single manufacturer (PFC Sigma; DePuy) were used in all TKAs. Patella resurfacing was not performed.

### Assessment of frailty

Frailty was measured based on the five criteria of the Physical Frailty Phenotype (PFP) proposed by Fried [3, 13], adapted as follows: shrinking (self-reported unintentional weight loss of more than 4,5 kg in the past year), exhaustion (self-reported using the CES-D depression scale), slowed walking speed (walking time of 5 m below an adjusted cut-off by gender and height), weakness [grip strength below an established cut-off based on gender and body mass index (BMI) measured on the dominant hand using a dynamometer (Jamar® Hydraulic Hand Dynamometer; Performance Health, Wisconsin)] and low physical activity (kilocalories per week below an established gender-specific cut-off using self-reported frequency and duration of walking or cycling based on activity level according to the Swiss Health Observatory). Each component or question was given a score of 0 or 1, depending on whether it was present or not. Robust patients were defined with a score of 0, pre-frail with a score of 1–2 and frail with a score of 3 and higher.

### Additional study variables

The *Charlson Comorbidity Index (CCI)* predicts the mortality of a patient who has a number of comorbidities, such as heart disease, AIDS or cancer (taking into account a total of 19 diseases). A value of zero means that no comorbidities were found; the higher the value, the higher the predicted mortality rate [14].

The purpose of the *Nutritional Risk Screening (NRS)* system is to detect the presence of malnutrition and the risk of developing malnutrition in the hospital setting. It includes four questions as a pre-screening. If one of these is answered positively, a screening follows which includes surrogate measures of nutritional status, with static and dynamic parameters and data on the severity of the disease (stress metabolism). For each parameter, a score from 0 to 3 can result. Age over 70 years is considered as a risk factor, and is included in the screening tool as well, giving 1 point. A total score of 3 or more points means that the patient is at risk of malnutrition or already malnourished and therefore a nutritional therapy is indicated [15].

The *Barthel Scale/Index* is an ordinal scale used to measure performance in activities of daily living (ADL). Ten variables describing ADL and mobility are scored, a higher number reflecting greater ability to function independently following hospital discharge [16].

The *Lawton & Brody Instrumental Activities of Daily Living Scale (IADL)* is an appropriate instrument to assess independent living skills. These skills are considered more complex than the basic activities of daily living as measured by the Barthel Index. There are eight domains of function measured with the Lawton IADL scale. Participants are scored according to their highest level of functioning in that category. A summary score ranges from 0 (low function, dependent) to 8 (high function, independent) [17].

*Short Physical Performance Battery (SPPB)* is a measure of physical functioning. SPPB evaluates balance, mobility, and muscle strength by examining an individual’s ability to stand in different positions, time to walk 4 m, and time to rise up from and sit down on a chair 5 times. The tests are scored between 0 and 4, leaving a maximum score of 12 [18].

The *Mini Mental State Examination (MMSE)* is a 30-point questionnaire that is used extensively in clinical and research settings to measure cognitive impairment. The test examines functions such as registration (repeating named prompts), attention and calculation, recall, language, ability to follow simple commands and orientation. Any score of 24 or more (out of 30) indicates a normal cognition. Below this, scores can indicate severe (≤ 9 points), moderate (10–18 points) or mild (19–23 points) cognitive impairment [19].

The 15-item *Geriatric Depression Scale (GDS-15)* is a short form of GDS and is used to screen, diagnose, and evaluate depression in elderly individuals. In scoring the GDS, 1 point is awarded for each answer that indicates depression. If a person scores more than 5 on the 15-question assessment, this may indicate the presence of depression [20].

### Statistical analysis

Descriptive information including demographic and morbidity-related characteristics were calculated for the whole sample. As a core statistical method one-way ANOVA with repeated measures was employed, to test whether there are significant differences between the four times of measurement in the study population, with the Fried Frailty Phenotype being the variable of interest and our primary outcome. If necessary, Greenhouse-Geisser correction was applied, to adjust for violation of the sphericity assumption. For this the R-package “afex” was used. If the repeated measures ANOVA yielded significant results and to examine differences between each pair of time of measurement post-hoc tests were performed using the “emmeans” R-package. In this procedure Bonferroni-correction was used to reduce the risk of a type I error. Further analyses included repeated measures ANOVA as well as post-hoc tests for the subgroups of pre-frail (Score 1–2) and frail patients (Score ≥ 3) and for each of the five subdomains (weight loss, exhaustion, low physical activity, slowness, weakness) of the Fried frailty phenotype as outcome variable. To examine whether certain patient characteristics can serve as predictors for improvement in frailty after joint replacement, we performed a logistic regression. For this a dichotomous variable which indicated whether a patient experienced any improvement in frailty after joint replacement was used as the dependent variable. For independent variables, we included age, gender, BMI, SPPB-Score, GDS-Score, and NRS-Score. All analyses were conducted in R version 4.2.1. *P*-values *p* < 0.05 were considered statistically significant.

## Results

### Baseline characteristics

Of a total of 101 participants, 50 were pre-frail (1–2 PFP criteria) and 51 were frail (≥ 3 PFP criteria) preoperatively. The female gender was much more prevalent in the total population at 70.30% and most prevalent in the frail cohort at 82.35%. Mean age at 80.04 ± 4.33 years, mean BMI at 29.86 ± 5.16 kg/m², mean number of medications at 8.29 ± 3.67, mean Charlson Comorbidity Index at 5.73 ± 1.89 and mean GDS-15 score at 4.06 ± 2.89 were higher in the frail participants. Mobility (mean SPPB score 5.00 ± 2.08) and (instrumental) activities of daily living (mean IADL score 6.14 ± 1.85, mean Barthel Index 88.24 ± 15.90) were most clearly reduced in the frail group (Table 1).

**Table 1:** Baseline characteristics

Characteristics	Total (*n* = 101)	Pre-frail (*n* = 50)	Frail (*n* = 51)
Female n (%)	71 (70.30)	29 (58.00)	42 (82.35)
Age y, mean ± SD	78.52 ± 4.51	76.98 ± 4.20	80.04 ± 4.33
BMI kg/m² mean ± SD	28.99 ± 4.74	28.10 ± 4.14	29.86 ± 5.16
Medication n mean ± SD	7.70 ± 3.78	7.10 ± 3.82	8.29 ± 3.67
Comorbidities n mean ± SD	7.53 ± 3.13	7.50 ± 2.98	7.57 ± 3.31
CCI mean ± SD	5.45 ± 1.94	5.16 ± 1.96	5.73 ± 1.89
NRS score (0–7) mean ± SD	1.28 ± 0.78	1.20 ± 0.61	1.35 ± 0.91
Barthel Index (0-100) mean ± SD	92.03 ± 12.47	95.90 ± 5.41	88.24 ± 15.90
IADL score (0–8) mean ± SD	6.69 ± 1.67	7.26 ± 1.24	6.14 ± 1.85
SPPB score (0–12) mean ± SD	6.66 ± 2.64	8.36 ± 2.01	5.00 ± 2.08
MMSE score (0–30) mean ± SD	26.95 ± 2.34	27.22 ± 2.25	26.69 ± 2.42
GDS-15 score (0–15) mean ± SD	3.49 ± 2.92	2.90 ± 2.87	4.06 ± 2.89

BMI, Body Mass Index; CCI, Charlson Comorbidity Index; NRS, Nutritional Risk Screening; IADL, Instrumental Activities of Daily Living; SPPB, Short Physical Performance Battery; MMSE, Mini-Mental State Examination; GDS, Geriatric Depression Scale.

### General improvement in frailty after THA/TKA

Hip or knee replacement improved PFP at 3 months in 80 patients (80%) from a pre-frail or frail status before surgery. In 14 participants (14%), PFP remained unchanged 3 months after joint replacement and in 6 patients (6%) PFP worsened.

### Frailty scores at the different measurement times

Table 2 demonstrates the total PFP scores for the whole sample (THA/TKA), the THA subgroup and the TKA subgroup, as well as the PFP scores of the prefrail and frail of the corresponding cohort.

**Fig. 1:**
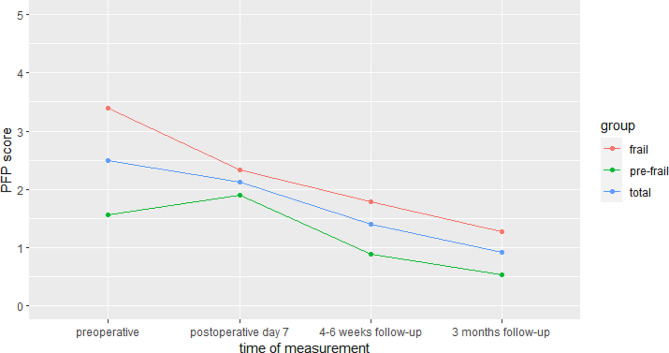
Physical Frailty Phenotype scores (means) of the total study group (*n* = 101), the pre-frail group (*n* = 50) and the frail group (*n* = 51) at the four different measurement points of the study

The mean PFP score of the total populations and the frail groups decreased continuously after surgery. In the pre-frail participants, the PFP score initially increased on postoperative day 7 before decreasing from 4 to 6 weeks follow-up. Frail patients benefited most from hip or knee replacements. Here, the mean PFP score decreased from 3.39 ± 1.45 preoperatively to 1.27 ± 1.14 3 months after surgery (*p* < 0.001) ). The course of the frailty scores of the total study group is shown graphically in Fig. 1.

**Table 2:** PFP score (mean ± SD) at different study time points of 101 participants

**Total sample (Hip and knee patients)**	**Total (** ***n*** ** = 101)**	**Pre-frail (** ***n*** ** = 50)**	**Frail (** ***n*** ** = 51)**
Frailty score pre-op (t0)	2.49 ± 1.05	1.56 ± 0.50	3.39 ± 1.45
Frailty score d 7 post-op (t1)	2.12 ± 1.43	1.90 ± 1.40	2.33 ± 1.44
Frailty score 4–6 wk follow-up (t2)	1.39 ± 1.39	0.88 ± 1.17	1.78 ± 1.45
Frailty score 12 wk follow-up (t3)	0.91 ± 1.17	0.53 ± 0.73	1.27 ± 1.14
**Hip patients**	**Total (** ***n*** ** = 65)**	**Pre-frail (** ***n*** ** = 26)**	**Frail (** ***n*** ** = 39)**
Frailty score pre-op (t0)	2.63 ± 1.02	1.54 ± 0.51	3.36 ± 0.49
Frailty score d 7 post-op (t1)	2.15 ± 1.46	2.11 ± 1.56	2.18 ± 1.41
Frailty score 4–6 wk follow-up (t2)	1.44 ± 1.44	1.08 ± 1.44	1.71 ± 1.39
Frailty score 12 wk follow-up (t3)	0.85 ± 1.09	0.50 ± 0.86	1.08 ± 1.18
**Knee patients**	**Total (** ***n*** ** = 36)**	**Pre-frail (** ***n*** ** = 24)**	**Frail (** ***n*** ** = 12)**
Frailty score pre-op (t0)	2.22 ± 1.05	1.58 ± 0.50	3.50 ± 0.52
Frailty score d 7 post-op (t1)	2.06 ± 1.39	1.67 ± 1.20	2.83 ± 1.47
Frailty score 4–6 wk follow-up (t2)	1.11 ± 1.28	0.65 ± 0.71	2.00 ± 1.65
Frailty score 12 wk follow-up (t3)	1.03 ± 1.32	0.57 ± 0.73	1.92 ± 1.73

PFP, Physical Frailty Phenotype; SD, Standard deviation; wk, week.

### Results of rmANOVA tests

Table 3 shows the time points between which there were statistically significant differences in the PFP scores of THA/TKA patients combined. In the total population and in the pre-frail group, there were no significant differences in PFP score preoperatively compared to 7 days postoperatively (hospital discharge) (*p* = 0.135 and *p* = 0.749). In addition, there was no significant difference in the PFP score between 4 and 6 weeks and 3 months follow-up in the pre-frail group (*p* = 0.136). There were significant differences between all other measures of PFP score in the rmANOVA tests in the total population as well as in the pre-frail and frail groups. As a result, the PFP score was significantly reduced in the pre-frail and frail groups 3 months after surgery (*p* < 0.001). In contrast to the pre-frail participants, the frail cohort benefited significantly from joint replacement as early as 7 days after surgery (*p* = 0.749 vs. *p* < 0.001). Separate repeated measures ANOVAs were also conducted for the THA and TKA subgroups. These were further subdivided into pre-frail and frail (Additional file 2).

**Table 3:** Results of rmANOVA tests for the four measurement points (t0-t3) of the Fried Frailty Phenotype

**Total sample**					
**Effect**	**df**	**MSE**	**F**	**ges**	***p*** **.value**
Time	2.61, 255.95	0.99	58.78	0.193	**< 0.001**
**contrast**	**estimate**	**SE**	**df**	**t.ratio**	***p*** **.value**
pre-op (t0) - d7 post-op (t1)	0.364	0.157	98	2.317	0.135
pre-op (t0) − 4–6 wk post-op (t2)	1.152	0.136	98	8.484	**< 0.001**
pre-op (t0) − 12 wk post-op (t3)	1.566	0.124	98	12.663	**< 0.001**
d7 post-op (t1) − 4–6 wk post-op (t2)	0.788	0.124	98	6.367	**< 0.001**
d7 post-op (t1) − 12 wk post-op (t3)	1.202	0.143	98	8.415	**< 0.001**
4–6 wk post-op (t2) − 12 wk post-op (t3)	0.414	0.103	98	4.039	**< 0.001**
*P* value adjustment: bonferroni method for 6 tests					
**Pre-frail**					
**Effect**	**df**	**MSE**	**F**	**ges**	***p*** **.value**
Time	2.31, 110.87	0.95	25.36	0.215	**< 0.001**
**contrast**	**estimate**	**SE**	**df**	**t.ratio**	***p*** **.value**
pre-op (t0) - d7 post-op (t1)	-0.327	0.209	48	-1.562	0.749
pre-op (t0) − 4–6 wk post-op (t2)	0.673	0.183	48	3.680	**0.004**
pre-op (t0) − 12 wk post-op (t3)	1.020	0.135	48	7.549	**< 0.001**
d7 post-op (t1) − 4–6 wk post-op (t2)	1.000	0.152	48	6.600	**< 0.001**
d7 post-op (t1) − 12 wk post-op (t3)	1.347	0.197	48	6.844	**< 0.001**
4–6 wk post-op (t2) − 12 wk post-op (t3)	0.347	0.147	48	2.354	0.136
*P* value adjustment: bonferroni method for 6 tests					
**Frail**					
**Effect**	**df**	**MSE**	**F**	**ges**	***p*** **.value**
Time	2.71, 132.75	0.92	49.06	0.286	**< 0.001**
**contrast**	**estimate**	**SE**	**df**	**t.ratio**	***p*** **.value**
pre-op (t0) - d7 post-op (t1)	1.04	0.192	49	5.429	**< 0.001**
pre-op (t0) − 4–6 wk post-op (t2)	1.62	0.178	49	9.092	**< 0.001**
pre-op (t0) − 12 wk post-op (t3)	2.10	0.177	49	11.884	**< 0.001**
d7 post-op (t1) − 4–6 wk post-op (t2)	0.58	0.192	49	3.023	**0.024**
d7 post-op (t1) − 12 wk post-op (t3)	1.06	0.207	49	5.125	**< 0.001**
4–6 wk post-op (t2) − 12 wk post-op (t3)	0.48	0.144	49	3.344	**0.010**
*P* value adjustment: bonferroni method for 6 tests					

df, degrees of freedom; MSE, mean squared error; SE, standard error; ges, generalised eta squared.

### Impact on frailty stages

**Fig. 2:**
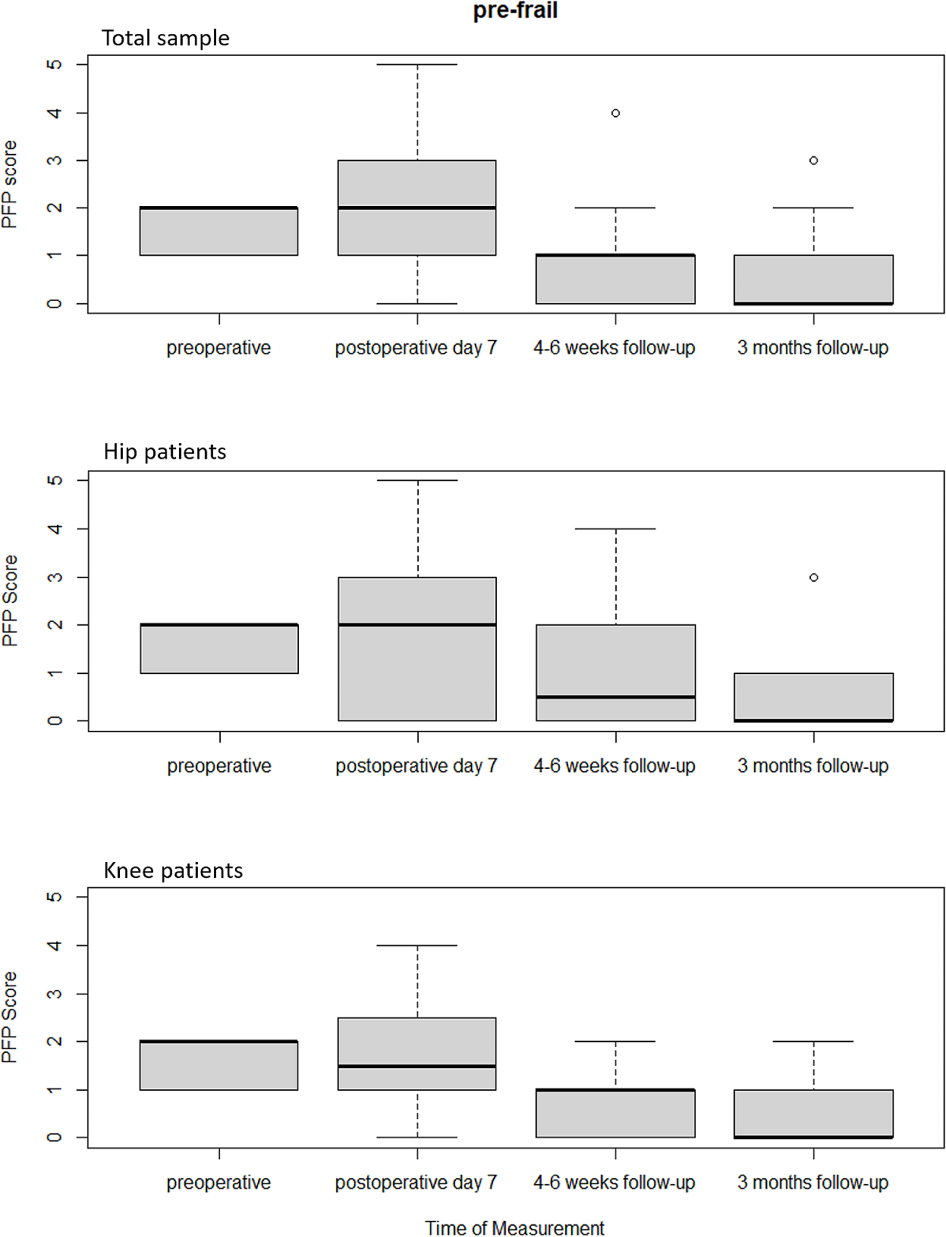
Box plots of the frailty scores according to Fried’s phenotype of the pre-frail groups pre- and postoperatively

Changes in frailty stages according to Fried can be well assessed by comparing the median values. Before hip or knee replacement, the median PFP score of the total sample in the pre-frail group was 2. Already 4–6 weeks postoperatively, the PFP score decreased to a median value of 1. 3 months after joint replacement, the median was 0. According to Fried’s criteria, pre-frailty was no longer present (Fig. 2).

**Fig. 3:**
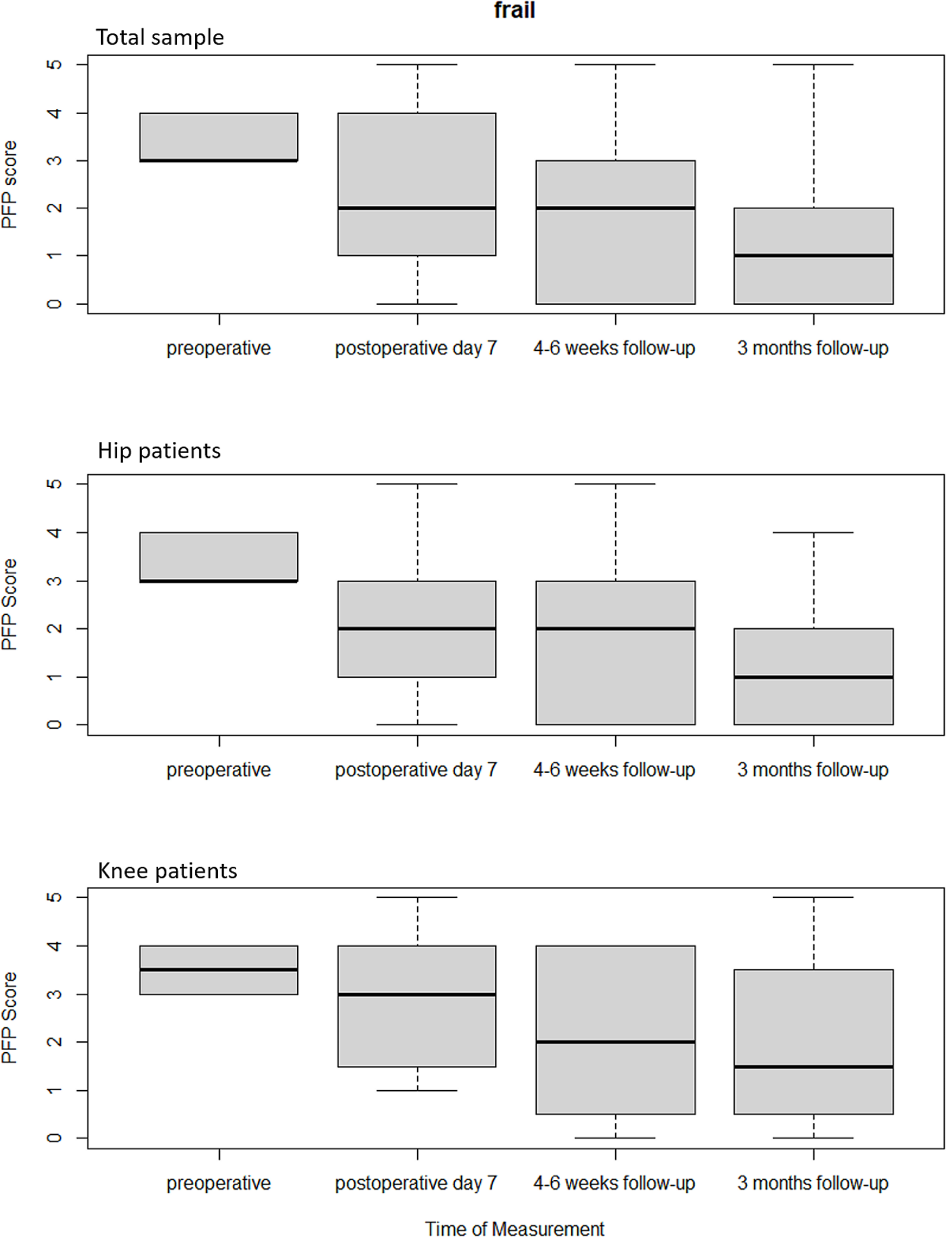
Box plots of the frailty scores according to Fried’s Phenotype of the frail groups pre- and postoperatively

The median PFP score in the frail cohort of the total sample before surgery was 3. Here, there was already a decrease in the median PFP score to 2 on postoperative day 7. 3 months after hip or knee replacement, the median PFP score was only 1, so that there were no longer any criteria for frailty according to Fried’s Phenotype (Fig. 3).

### Fried frailty phenotype subscores for the total study group (THA/TKA)

The results of the repeated measures ANOVA tests for each of the five subscores of the Fried Frailty Phenotype (weight loss, exhaustion, slowness, weakness, low physical activity) of the total population, the prefrail and frail group can be found in Additional file 1. While no significant differences were found for the two criteria weight loss and weakness (grip strength), significant differences were shown in the subscores exhaustion, slow walking speed and low physical activity between different time points of the measurement. This applies to the total population, but also to pre-frail and frail cohorts.

### Exclusion of confounding factors in the improvement of frailty

Logistic regression analysis (univariate and multivariate) was used to determine whether the variables “Age”, “Gender”, “BMI”, “SPPB”, “GDS” and “NRS” had an influence on the improvement in frailty between t0 (pre-OP) and t3 (12 wk follow-up). As can be seen in Table 4, this was not the case for any of the variables included. Thus, we assume that there were no confounding factors. It can therefore be concluded that joint replacement can improve frailty in patients independent of the analysed characteristics.

**Table 4:** Logistic Regression on predictors of improvement in frailty after THA/TKA

	Univariate	Multivariate
(Intercept)		1.65 ***
		[0.99, 2.31]
Age	-0.10	-0.04
	[-0.60, 0.39]	[-0.60, 0.52]
Gender (0 = female)	-0.56	-0.65
	[-1.58, 0.46]	[-1.78, 0.49]
BMI	0.13	-0.03
	[-0.38, 0.63]	[-0.60, 0.54]
SPPB-Score	0.10	0.04
	[-0.39, 0.60]	[-0.60, 0.68]
GDS-Score	-0.38	-0.38
	[-0.83, 0.07]	[-0.90, 0.14]
NRS-Score	-0.03	0.01
	[-0.51, 0.45]	[-0.52, 0.54]

The outcome was a dichotomous variable indicating whether there was an improvement in frailty between t0 and t3. *** *p* < 0.001; ** *p* < 0.01; * *p* < 0.05.

## Discussion

### Purpose of the study

Frailty has mainly been considered as a predictor of adverse events after surgery. There are several studies on this in the field of arthroplasty [21–23]. For example, Meyer et al. described a higher rate of reoperations, hospital readmissions, surgical and non-surgical complications, and blood transfusions in elderly frail patients [21]. In Johnson et al. frailty was associated with increased perioperative complication rates and mortality [23]. This should be considered before indicating THA/TKA, especially if geriatric co-management is not possible. Risk stratification is of great importance here. However, little is known about the impact of hip and knee replacement in OA on frailty. OA is considered the most prevalent chronic joint disease in the world and has a particularly high mortality rate when combined with frailty [7, 24]. Some publications have shown a strong, independent association between OA and pre-frailty/frailty in people aged 65 years and older [6, 25, 26]. The purpose of this prospective study was to show that in elderly patients with OA, primary hip or knee replacement can have a significant impact on the regression or reversibility of frailty. The results suggest that THA and TKA are effective interventions for the prevention and treatment of pre-frailty and frailty in older patients with osteoarthritis. Participants with pre-frailty and a median PFP score of 2 preoperatively had a median PFP score of 0 3 months after THA/TKA and were robust. In the frail patients with a preoperative medial PFP score of 3, the medial PFP score decreased to 1 after surgery. The patients were no longer in a frail condition.

### Interventions for frailty

Due to the high prevalence of OA and frailty in people aged 65 years and older, the strong association between the two processes, and the fact that frailty is a predictor of increased mortality in people with OA [24], there is an international call for preventive and therapeutic interventions [6]. However, little is known about the reversibility of pre-frailty and frailty. So far, interventions have mainly been limited to physical training, high-protein diets, or a combination of both [9]. These conservative interventions have been studied and applied in pre-frail/frail patients without the context of OA and indicated THA or TKA. However, this type of intervention may play a role in the future before surgery (prehabilitation) to reduce the risk of surgery. Frailty can be influenced and is therefore a modifiable preoperative risk factor that is associated with some adverse events [21, 23]. Currently, there is only good evidence for physical training and the combination of physical training and high-protein diet [9].

The results of previous interventions to delay the progression of frailty or improve frailty are sometimes very limited [9]. Also, many studies focus on pre-frail patients. In 2018, Gené Huguet et al. achieved a significant return from pre-frailty to robustness through a six-month interdisciplinary intervention based on physical activity, Mediterranean dietary counselling, an assessment of inappropriate prescribing in patients with polypharmacy and a social assessment. Frail patients were not included [27]. This result could also be achieved in our study by a THA or TKA. However, hip or knee replacement surgery has also been shown to significantly reduce the stage of existing frailty.

### Reversing frailty in older adults

Frailty probably seems to be more influenceable than previously assumed. However, further studies are certainly needed on this. Factors that may trigger or exacerbate frailty, but also lead to regression or even reversibility, should be explored. The recently published study by Kolle et al. emphasises the topicality and relevance of the study results in the context of reversing frailty. The approach in our work is in line with the current understanding of the reversal of frailty [28]. Despite the growing importance of frailty, there is still no international consensus on a uniform definition and assessment. The frailty score proposed by Fried et al. [9] is suitable for prospective studies and intervention evaluation. In addition, PFP has biological validity and is easy and inexpensive to measure [29].

### Limitations

This study has some limitations. The study is part of the ongoing SOG trial and the analyses presented here were not originally planned. For this reason, there is no control group. Although the participants had exhausted all conservative measures (analgesics including opioids, physiotherapy and often rehabilitation) as a prerequisite for the surgery, there are always circumstances that could have influenced the frailty even without special intervention. This could include, for example, psychosocial aspects or the treatment of comorbidities. However, as previous studies have shown, interventions are usually needed to achieve regression or even reversibility of pre-/frailty. Spontaneous improvements are hardly to be expected [9]. This is a single-centre study with possible limitations in the heterogeneity of the study population and potential ‘centre bias’. Whether and to what extent geriatric co-management provides additional benefits in improving frailty, especially after 3 months, is unknown. No data are available on this. This is being investigated for the first time in the still ongoing SOG study. Strictly considered, there are some factors postoperatively that could possibly play a role beyond that, e.g. surgery according to the fast-track principle, duration and type of rehabilitation or postoperative complications. Here, however, one comes up against ethical limits. It will not be possible to refuse a patient geriatric co-management or a certain rehabilitation if it is necessary. On the contrary, these procedures are necessary to regain joint function.

The fact that preoperatively all conservative treatments (physical training, etc.) were exhausted and after 3 months postoperatively the frailty status improved in 80% of the participants strongly suggests the benefit of hip/knee replacement.

### Strengths

A major strength of this study is its prospective design with 101 participants. Studies on interventions for frailty often have a small number of participants. It is very difficult to recruit older people with frailty to take part in a trial. In previous studies, the proportion of participants with pre-frailty often predominated. Many trials included people who were either pre-frail or frail. In this study, even slightly more patients with frailty could be included. Separate analyses were performed for total, pre-frail and frail participants. The study population has a variety of risk factors for frailty such as advanced age, female gender, obesity, multimorbidity, malnutrition, polypharmacy, and reduced mobility according to Hoogendijk et al. [1] (Table 1). There were few drop-outs and few patients were lost to follow-up. Data analysis was performed externally and independently by the Department of Health Economics at the Technical University of Munich.

Identifying frailty as a predictor of adverse events is important. But pre-frailty and frailty should also be perceived as a risk factor that can be modified. With appropriate interventions, both can be improved or reversed. It may be possible not only to achieve functional improvement, but also to improve prognosis and reduce mortality. Therefore, further studies on interventions for frailty are needed.

## Conclusion

Primary total hip and knee arthroplasty results in a significant decrease in frailty score in older patients with OA as measured by the Fried Frailty Phenotype. Pre-frail participants were often robust after joint replacement. Frailty could be improved by THA or TKA to the pre-frailty stage, so that no further frailty was present. In conclusion, joint replacement can be seen as an effective intervention for the prevention and treatment of frailty in patients with hip and knee osteoarthritis.

### Electronic supplementary material

Below is the link to the electronic supplementary material.

Additional file 1. Repeated measures ANOVA for the 5 subscores of the Fried Frailty Phenotype

Additional file 2. Repeated measures ANOVA for the THA and TKA subgroups

## Data Availability

The data are available on reasonable request from the corresponding author.

## Abbreviations

ANOVA: Analysis of variance
BMI: Body Mass Index
CCI: Charlson Comorbidity Index
df: degrees of freedom
GDS: Geriatric Depression Scale
ges: generalised eta squared
IADL: Instrumental Activities of Daily Living
MMSE: Mini-Mental State Examination
MSE: Mean squared error
NRS: Nutritional Risk Screening
OA: Osteoarthritis
PFP: Physical Frailty Phenotype
SD: Standard deviation
SE: Standard error
SOG: Special Orthopaedic Geriatrics
SPPB: Short Physical Performance Battery
THA: Total hip arthroplasty
TKA: Total knee arthroplasty
wk: week
