# Supplementary material for: The impact of primary total hip and knee replacement on frailty: an observational prospective analysis
Source: BMC Musculoskelet Disord. 2024 Jan 20;25:78. doi: 10.1186/s12891-024-07210-w (PMC10799496; doi:10.1186/s12891-024-07210-w)
Supplement: Supplementary file 1 — Additional file 1. Repeated measures ANOVA for the 5 subscores of the Fried Frailty Phenotype [file 12891_2024_7210_MOESM1_ESM.pdf]

Additional file 1

Supplementary file contents:

These further analyses included repeated measures ANOVA as well as post-hoc tests for the subgroups of the total sample, pre-frail (score 1-2) and frail patients (score ≥ 3), and for each of the five subdomains (weight loss, exhaustion, slowness, weakness, low physical activity) of the Fried Frailty Phenotype as an outcome variable.

Supplementary Table 1 rmANOVA Subscore Weight loss

**Total sample**					
**Effect**	**df**	**MSE**	**F**	**ges**	**p.value**
Time	2.62, 253.69	0.06	44652	.006	.371
**Pre-frail**					
**Effect**	**df**	**MSE**	**F**	**ges**	**p.value**
Time	2.29, 109,68	0.03	0.73	0.12	0.502
**Frail**					
**Effect**	**df**	**MSE**	**F**	**ges**	**p.value**
Time	2.51, 120.56	0.08	2.55	.030	.069

Supplementary Table 2 rmANOVA Subscore Exhaustion

**Total sample**					
**Effect**	**df**	**MSE**	**F**	**ges**	**p.value**
Time	2.72, 264.13	0.16	49.40	.223	**<.001**
**contrast**	**estimate**	**SE**	**df**	**t.ratio**	**p.value**
pre-op (t0) - d7 post-op (t1)	0.337	0.0648	97	5198	**<.001**
pre-op (t0) - 4-6 wk post-op (t2)	0.490	0.0547	97	8947	**<.001**
pre-op (t0) - 12 wk post-op (t3)	0.633	0.0511	97	12392	**<.001**
d7 post-op (t1) - 4-6 wk post-op (t2)	0.153	0.0510	97	3002	**0.020**
d7 post-op (t1) - 12 wk post-op (t3)	0.296	0.0566	97	5231	**<.001**
4-6 wk post-op (t2) - 12 wk post-op (t3)	0.143	0.0481	97	2970	**0.023**
P value adjustment: bonferroni method for 6 tests					
**Pre-frail**					
**Effect**	**df**	**MSE**	**F**	**ges**	**p.value**
Time	2.47, 118.49	0.19	22.41	.230	**<.001**
**contrast**	**estimate**	**SE**	**df**	**t.ratio**	**p.value**
pre-op (t0) - d7 post-op (t1)	0.1837	0.0996	48	1844	0.429
pre-op (t0) - 4-6 wk post-op (t2)	0.4898	0.0778	48	6294	**<.001**
pre-op (t0) - 12 wk post-op (t3)	0.5714	0.0714	48	8000	**<.001**
d7 post-op (t1) - 4-6 wk post-op (t2)	0.3061	0.0726	48	4215	**<.001**
d7 post-op (t1) - 12 wk post-op (t3)	0.3878	0.0866	48	4478	**<.001**
4-6 wk post-op (t2) - 12 wk post-op (t3)	0.0816	0.0641	48	1273	0.212
P value adjustment: bonferroni method for 6 tests					
**Frail**					
**Effect**	**df**	**MSE**	**F**	**ges**	**p.value**
Time	2.89, 138.73	0.13	32.84	.263	**<.001**
**contrast**	**estimate**	**SE**	**df**	**t.ratio**	**p.value**
pre-op (t0) - d7 post-op (t1)	0.490	0.0778	48	6.294	**<.001**
pre-op (t0) - 4-6 wk post-op (t2)	0.490	0.0778	48	6.294	**<.001**
pre-op (t0) - 12 wk post-op (t3)	0.694	0.0726	48	9.553	**<.001**
d7 post-op (t1) - 4-6 wk post-op (t2)	0.000	0.0652	48	0.000	1.000
d7 post-op (t1) - 12 wk post-op (t3)	0.204	0.0713	48	2.862	**0.037**
4-6 wk post-op (t2) - 12 wk post-op (t3)	0.204	0.0713	48	2.862	**0.037**
P value adjustment: bonferroni method for 6 tests					

Supplementary Table 3 rmANOVA Subscore Slowed walking speed

**Total sample**					
**Effect**	**df**	**MSE**	**F**	**ges**	**p.value**
Time	2.84, 275.56	0.15	33.09	.140	**<.001**
**contrast**	**estimate**	**SE**	**df**	**t.ratio**	**p.value**
pre-op (t0) - d7 post-op (t1)	-0.173	0.0543	97	-3193	**0.011**
pre-op (t0) - 4-6 wk post-op (t2)	0.173	0.0562	97	3085	**0.016**
pre-op (t0) - 12 wk post-op (t3)	0.327	0.0538	97	6065	**<.001**
d7 post-op (t1) - 4-6 wk post-op (t2)	0.347	0.0545	97	6369	**<.001**
d7 post-op (t1) - 12 wk post-op (t3)	0.500	0.0548	97	9132	**<.001**
4-6 wk post-op (t2) - 12 wk post-op (t3)	0.153	0.0444	97	3451	**0.005**
P value adjustment: bonferroni method for 6 tests					
**Pre-frail**					
**Effect**	**df**	**MSE**	**F**	**ges**	**p.value**
Time	2.43, 116.77	0.15	21.34 ***	.207	**<.001**
**contrast**	**estimate**	**SE**	**df**	**t.ratio**	**p.value**
pre-op (t0) - d7 post-op (t1)	-0.4286	0.0772	48	-5555	**<.001**
pre-op (t0) - 4-6 wk post-op (t2)	-0.0204	0.0743	48	-0.275	1000
pre-op (t0) - 12 wk post-op (t3)	0.0816	0.0641	48	1273	1000
d7 post-op (t1) - 4-6 wk post-op (t2)	0.4082	0.0767	48	5322	**<.001**
d7 post-op (t1) - 12 wk post-op (t3)	0.5102	0.0778	48	6556	**<.001**
4-6 wk post-op (t2) - 12 wk post-op (t3)	0.1020	0.0437	48	2335	0.143
P value adjustment: bonferroni method for 6 tests					
**Frail**					
**Effect**	**df**	**MSE**	**F**	**ges**	**p.value**
Time	2.76, 132.40	0.14	25.98	.249	**<.001**
**Contrast**	**estimate**	**SE**	**df**	**t.ratio**	**p.value**
pre-op (t0) - d7 post-op (t1)	0.0816	0.0571	48	1.429	0.957
pre-op (t0) - 4-6 wk post-op (t2)	0.3673	0.0754	48	4.869	**0.001**
pre-op (t0) - 12 wk post-op (t3)	0.5714	0.0714	48	8.000	**<.001**
d7 post-op (t1) - 4-6 wk post-op (t2)	0.2857	0.0772	48	3.703	**0.003**
d7 post-op (t1) - 12 wk post-op (t3)	0.4898	0.0778	48	6.294	**<.001**
4-6 wk post-op (t2) - 12 wk post-op (t3)	0.2041	0.0770	48	2.649	0.065
P value adjustment: bonferroni method for 6 tests					

Supplementary Table 4 rmANOVA Subscore Weakness (grip strength)

**Total sample**					
**Effect**	**df**	**MSE**	**F**	**ges**	**p.value**
Time	2.80, 271.66	0.07	0.26	<.001	0.840
**Pre-frail**					
**Effect**	**df**	**MSE**	**F**	**ges**	**p.value**
Time	2.07, 99.28	0.06	1.41	.005	0.248
**Frail**					
**Effect**	**df**	**MSE**	**F**	**ges**	**p.value**
Time	2.82, 135.48	0.09	0.06	<.001	.978

Supplementary Table 5 rmANOVA Subscore Low physical activity

**Total sample**					
**Effect**	**df**	**MSE**	**F**	**ges**	**p.value**
Time	2.71, 262.45	0.19	38.52	.201	**<.001**
**contrast**	**estimate**	**SE**	**df**	**t.ratio**	**p.value**
pre-op (t0) - d7 post-op (t1)	0.255	0.0685	97	3723	**0.002**
pre-op (t0) - 4-6 wk post-op (t2)	0.469	0.0636	97	7384	**<.001**
pre-op (t0) - 12 wk post-op (t3)	0.582	0.0541	97	10746	**<.001**
d7 post-op (t1) - 4-6 wk post-op (t2)	0.214	0.0585	97	3665	**0.002**
d7 post-op (t1) - 12 wk post-op (t3)	0.327	0.0558	97	5856	**<.001**
4-6 wk post-op (t2) - 12 wk post-op (t3)	0.112	0.0479	97	2346	0.126
P value adjustment: bonferroni method for 6 tests					
**Pre-frail**					
**Effect**	**df**	**MSE**	**F**	**ges**	**p.value**
Time	2.53, 121.60	0.22	14.14	.177	**<.001**
**contrast**	**estimate**	**SE**	**df**	**t.ratio**	**p.value**
pre-op (t0) - d7 post-op (t1)	0.000	0.1010	48	0.000	1000
pre-op (t0) - 4-6 wk post-op (t2)	0.327	0.1026	48	3181	**0.015**
pre-op (t0) - 12 wk post-op (t3)	0.449	0.0828	48	5423	**<.001**
d7 post-op (t1) - 4-6 wk post-op (t2)	0.327	0.0845	48	3866	**0.002**
d7 post-op (t1) - 12 wk post-op (t3)	0.449	0.0718	48	6254	**<.0001**
4-6 wk post-op (t2) - 12 wk post-op (t3)	0.122	0.0692	48	1769	0.499
P value adjustment: bonferroni method for 6 tests					
**Frail**					
**Effect**	**df**	**MSE**	**F**	**ges**	**p.value**
Time	2.81, 135.09	0.14	36.98	.302	**<.001**
**contrast**	**estimate**	**SE**	**df**	**t.ratio**	**p.value**
pre-op (t0) - d7 post-op (t1)	0.510	0.0778	48	6556	**<.001**
pre-op (t0) - 4-6 wk post-op (t2)	0.612	0.0703	48	8706	**<.001**
pre-op (t0) - 12 wk post-op (t3)	0.714	0.0652	48	10954	**<.001**
d7 post-op (t1) - 4-6 wk post-op (t2)	0.102	0.0785	48	1300	1000
d7 post-op (t1) - 12 wk post-op (t3)	0.204	0.0824	48	2478	0.101
4-6 wk post-op (t2) - 12 wk post-op (t3)	0.102	0.0668	48	1528	0.799
P value adjustment: bonferroni method for 6 tests					

df, degrees of freedom; MSE, mean squared error; SE, standard error; ges, generalised eta squared
