# Supplementary material for: The impact of primary total hip and knee replacement on frailty: an observational prospective analysis
Source: BMC Musculoskelet Disord. 2024 Jan 20;25:78. doi: 10.1186/s12891-024-07210-w (PMC10799496; doi:10.1186/s12891-024-07210-w)
Supplement: Supplementary file 2 — Additional file 2. Repeated measures ANOVA for the THA and TKA subgroups [file 12891_2024_7210_MOESM2_ESM.pdf]

Additional file 2

Supplementary file contents:

These further analyses included repeated measures ANOVA for the THA and TKA subgroups. These were further subdivided into pre-frail and frail.

Supplementary Table 1 Results of the rmANOVA subanalysis of the THA group for the four measurement points (t0-t3) of the Fried Frailty Phenotype

**Hip patients**					
**Pre-frail**					
**Effect**	**df**	**MSE**	**F**	**ges**	**p.value**
Time	2.04, 50.97	1.20	14.96	.211	**<.001**
**contrast**	**estimate**	**SE**	**df**	**t.ratio**	**p.value**
pre-op (t0) - d7 post-op (t1)	-0.577	0.315	25	-1.834	0.472
pre-op (t0) - 4-6 wk post-op (t2)	0.462	0.289	25	1.594	0.740
pre-op (t0) - 12 wk post-op (t3)	1.038	0.180	25	5.783	**<.001**
d7 post-op (t1) - 4-6 wk post-op (t2)	1.038	0.204	25	5.099	**<.001**
d7 post-op (t1) - 12 wk post-op (t3)	1.615	0.272	25	5.935	**<.001**
4-6 wk post-op (t2) - 12 wk post-op (t3)	0.577	0.216	25	2.670	0.079
P value adjustment: bonferroni method for 6 tests					
**Frail**					
**Effect**	**df**	**MSE**	**F**	**ges**	**p.value**
Time	2.43, 89.83	0.94	46.08	.337	**<.001**
**contrast**	**estimate**	**SE**	**df**	**t.ratio**	**p.value**
pre-op (t0) - d7 post-op (t1)	1.158	0.212	37	5.468	**<.001**
pre-op (t0) - 4-6 wk post-op (t2)	1.658	0.186	37	8.922	**<.001**
pre-op (t0) - 12 wk post-op (t3)	2.263	0.167	37	13.525	**<.001**
d7 post-op (t1) - 4-6 wk post-op (t2)	0.500	0.232	37	2.158	0.225
d7 post-op (t1) - 12 wk post-op (t3)	1.105	0.238	37	4.646	**<.001**
4-6 wk post-op (t2) - 12 wk post-op (t3)	0.605	0.149	37	4.071	**0.001**
P value adjustment: bonferroni method for 6 tests					

Supplementary Table 2 Results of the rmANOVA subanalysis of the TKA group for the four measurement points (t0-t3) of the Fried Frailty Phenotype

**Knee patients**					
**Pre-frail**					
**Effect**	**df**	**MSE**	**F**	**ges**	**p.value**
Time	2.56, 56.31	0.72	11.98	.269	**<0.001**
**contrast**	**estimate**	**SE**	**df**	**t.ratio**	**p.value**
pre-op (t0) - d7 post-op (t1)	-0.044	0.263	22	-0.165	1.000
pre-op (t0) - 4-6 wk post-op (t2)	0.913	0.208	22	4.396	**0.001**
pre-op (t0) - 12 wk post-op (t3)	1.000	0.209	22	4.796	**0.001**
d7 post-op (t1) - 4-6 wk post-op (t2)	0.957	0.231	22	4.144	**0.003**
d7 post-op (t1) - 12 wk post-op (t3)	1.044	0.277	22	3.761	**0.007**
4-6 wk post-op (t2) - 12 wk post-op (t3)	0.087	0.188	22	0.463	1.000
P value adjustment: bonferroni method for 6 tests					
**Frail**					
**Effect**	**df**	**MSE**	**F**	**ges**	**p.value**
Time	2.55, 28.00	1.27	6.26	.184	.003
**contrast**	**estimate**	**SE**	**df**	**t.ratio**	**p.value**
pre-op (t0) - d7 post-op (t1)	0.667	0.432	11	1.542	0.908
pre-op (t0) - 4-6 wk post-op (t2)	1.500	0.469	11	3.200	0.051
pre-op (t0) - 12 wk post-op (t3)	1.583	0.499	11	3.171	0.053
d7 post-op (t1) - 4-6 wk post-op (t2)	0.833	0.322	11	2.590	0.151
d7 post-op (t1) - 12 wk post-op (t3)	0.917	0.434	11	2.110	0.352
4-6 wk post-op (t2) - 12 wk post-op (t3)	0.083	0.358	11	0.233	1.000
P value adjustment: bonferroni method for 6 tests					
